# Gold Nanoparticles AuNP Decorated on Fused Graphene-like Materials for Application in a Hydrogen Generation

**DOI:** 10.3390/ma16134779

**Published:** 2023-07-03

**Authors:** Erik Biehler, Qui Quach, Tarek M. Abdel-Fattah

**Affiliations:** Applied Research Center, Thomas Jefferson National Accelerator Facility, Department of Molecular Biology and Chemistry, Christopher Newport University, Newport News, VA 23606, USA; erik.biehler.16@cnu.edu (E.B.); qui.quach.13@cnu.edu (Q.Q.)

**Keywords:** gold nanoparticles, fused graphene-like materials, composites, catalyst, hydrogen generation

## Abstract

The search for a sustainable, alternative fuel source to replace fossil fuels has led to an increased interest in hydrogen fuel. This combustible gas is not only clean-burning but can readily be produced via the hydrolysis of sodium borohydride. The main drawback of this reaction is that the reaction occurs relatively slowly and requires a catalyst to improve efficiency. This study explored a novel composite material made by combining gold nanoparticles and fused graphene-like materials (AuFGLM) as a catalyst for generating hydrogen via sodium borohydride. The novel fused graphene-like material (FGLM) was made with a sustainable dextrose solution and by using a pressure-processing method. Imaging techniques showed that FGLM appears to be an effective support template for nanoparticles. Transmission electron microscopy (TEM), scanning electron microscopy (SEM), energy-dispersive X-ray spectroscopy (EDS), Fourier-transform infrared spectroscopy (FTIR), X-ray diffraction (XRD) and Raman spectroscopy were used to characterize and determine the size, shape, and structure of nanoparticles and composites. The TEM study characterized the fused carbon backbone as it began to take on a rounder shape. The TEM images also revealed that the average diameter of the gold nanoparticle was roughly 23 nm. The FTIR study confirmed O-H, C-C, and C=O as functional groups in the materials. The EDS analysis showed that the composite contained approximately 6.3% gold by weight. The crystal structures of FGLM and AuFGLM were identified via P-XRD analysis. Various reaction conditions were used to test the catalytic ability of AuFGLM, including various solution pHs, temperatures, and doses of NaBH_4_. It was observed that optimal reaction conditions included high temperature, an acidic solution pH, and a higher dose of NaBH_4_. The activation energy of the reaction was determined to be 45.5 kJ mol^−1^, and it was found that the catalyst could be used multiple times in a row with an increased volume of hydrogen produced in ensuing trials. The activation energy of this novel catalyst is competitive compared to similar catalysts and its ability to produce hydrogen over multiple uses makes the material an exciting choice for catalyzing the hydrolysis of NaBH_4_ for use as a hydrogen fuel source.

## 1. Introduction

It is estimated that up to 84.3% of global energy comes from fossil fuels. These fossil fuels are not only dwindling, but their combustion is a leading cause of greenhouse gas emissions [1,2]. These issues have led to a search for an alternative energy source to replace the world’s dependence on fossil fuels. One promising option is that of hydrogen gas, which is not only the most abundant element in the universe but is also clean-burning, as it only produces water as a byproduct. Safety concerns regarding the storage of this highly combustible gas are standing in the way of the widespread implementation of hydrogen as a fuel source; thus, much work is currently being done to find a safer method of storage. One potential method is the use of a hydrogen feedstock material (HFM), particularly sodium borohydride (NaBH_4_), which contains 10.8% hydrogen by weight. NaBH_4_ also readily reacts in water, releasing gaseous hydrogen (1) [3].

NaBH_4_ + 2H_2_O → NaBO_2_ + 4H_2_ (1)

This reaction, however, occurs relatively slowly and requires a catalyst to improve the generation rate of hydrogen [3]. In recent years, precious metal nanoparticles have been utilized for a variety of different applications including gas detection [4], anti-bacterial properties [5], optoelectrical properties [6], as catalysts [7,8], and for hydrogen generation [9,10], including as a catalyst for the hydrolysis of NaBH_4_ [11,12,13,14]. Gold nanoparticles, for instance, are used as catalysts in various chemical reactions. Although gold nanoparticles are not as widely used as other transition metal nanoparticles, such as palladium or platinum, gold nanoparticles possess unique properties that make them valuable catalysts in specific applications. Gold nanoparticles exhibit excellent catalytic activity and selectivity, particularly in oxidation and reduction reactions [15,16,17,18,19]. They can effectively catalyze reactions involving small molecules, such as carbon monoxide, oxygen, hydrogen, and alcohols. The catalytic properties of gold nanoparticles are highly dependent on their size and shape [20]. By controlling these parameters during synthesis, researchers can tailor the catalytic performance of the nanoparticles for specific reactions. Gold nanoparticles can be synthesized using several methods, including chemical reduction, sol–gel processes, and colloidal synthesis [15,16,17,18,19,20]. Smaller-sized nanoparticles often exhibit higher catalytic activity, especially when catalyzing the hydrolysis of NaBH_4_. For example, in a study by Quach et al. (2021), the researchers synthesized gold nanoparticles with an average size of 8 nm and applied them as catalysts in a hydrogen generation reaction [11]. The catalyzing reactions achieved the highest reaction rate of pH 6, 303 K with 1225 µmoles of NaBH_4_ [11]. The gold nanoparticles successfully kept catalyzing the reaction up to the fifth trial and assisted the reaction in generating 28.9 mL of hydrogen [12].

It is important to note that one drawback of nanoparticles is their tendency to agglomerate in solutions, which can lead to a decrease in their total surface area and overall catalytic ability [21]. Therefore, these nanoparticle catalysts are often combined with a support structure. The support structure not only increases the stability of the nanoparticles but also lowers the required activation energy. For example, a study by Osborne et al. (2020) indicated that the activation energy of gold nanoparticles supporting over-activated carbon (21.6 kJ mol^−1^) was much lower than the unsupported nanoparticles (231.7 kJ mol^−1^) [22]. It is challenging to find a safe, effective, and economical supported material; however, various support templates have been explored, including carbon nanotubes (CNTs). Graphene and mesoporous silica have been used to stabilize and separate nanoparticles [23]. Carbon-based materials are often an ideal choice for support materials due to their unique properties, such as a high surface area and stability [21,22,23,24,25]. One example of a carbon-based material that could potentially be used as a support for nanoparticles is, graphene, a well-known material which has been applied in the fields of batteries, solar cells, sensors, and catalysis [23]. Graphene is among the thinnest materials, and possesses exceptional mechanical strength. Its structure contains two-dimensional sp^2^-hybridized carbon atom planar sheets stacked into honeycomb lattices. It was reported in study by Ghosh et al. that the surface area of graphene was higher than that of CNTs [26]. However, the production cost of pristine graphene is high, and its synthesis method is quite difficult [23]. Other graphene structures, including graphene oxide and reduced graphene oxide, have a lower production cost and easier preparation methods, but their methods of production often required toxic chemicals that pose a risk to human health [23,27]. In our study, we found a novel way to synthesize a fused graphene-like material (FGLM). The method was simple and only used dextrose, a renewable and eco-friendly material. The synthesized FGLM was then used as a support material in order to prevent gold nanoparticle agglomeration and improve their catalytic ability.

In this study, the reduction method was used to control the size, shape, and surface properties of the gold nanoparticles, positively influencing their catalytic behavior. Next, the fused graphene-like material was synthesized, characterized, and used as a support structure. The two synthesized materials were then combined to form the novel composite material, which was comprised of gold nanoparticles supported on fused graphene-like materials (AuFGLM). This new material was characterized and explored for its ability to catalyze the hydrolysis reaction of NaBH_4_. The AuFGLM material was characterized via transmission electron microscopy (TEM), energy dispersive spectroscopy (EDS), Fourier transform infrared spectroscopy (FTIR), X-ray diffraction (XRD), and Raman spectroscopy. Then, the AuFGLM was tested for its catalytic ability under different doses of NaBH_4_, at different temperatures, and pH values, and to determine its reusability.

## 2. Experimental

### 2.1. Synthesis of Gold Nanoparticles

We applied the method of synthesizing nanoparticles by described by Quach and Abdel-Fattah [5]. Gold nanoparticles were independently formed from the reduction of chloroauric acid (AuCl_4_H) using sodium citrate as a stabilizer. First a 1 mM precursor solution was prepared by dissolving the chloroauric acid in 100 mL of water. This solution was then heated until boiling and 1% w/w sodium citrate solution was added dropwise until a color change was observed. The solution was removed from the heat and allowed to cool to room temperature.

### 2.2. Synthesis of Fused Graphene-like Materials and Nanocomposites

To synthesize the fused graphene-like material, a 0.5 M dextrose solution was prepared by dissolving powdered dextrose into deionized water (18 MΩ). The dextrose solution was added to a stainless-steel reaction vessel in a ratio of 3:2, gas to liquid. The reaction vessel was then placed into an oven and heated at 200 °C for four hours to form the fused graphene-like materials (FGLM). The FGLM material was centrifuged and washed several times with deionized water (18 MΩ) and ethanol to remove any impurities. Once washed, the fused graphene-like materials were dried and stored for future use.

**Scheme 1:**
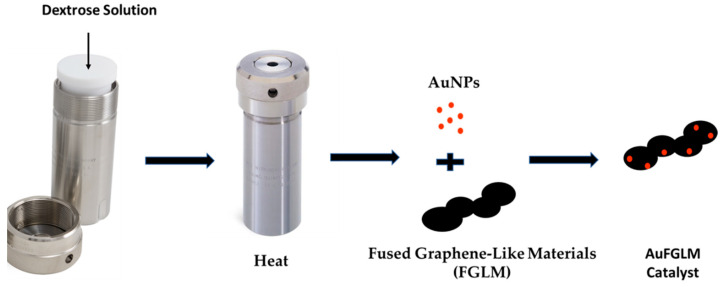
Illustration of AuFGLM composite synthesis. The black arrow depicted the step by step of the synthesis process andhe red dots represent gold nanoparticles (AuNPs).

The AuFGLM composites were produced by incipient wetness impregnation of roughly 300 mg FGLMs with 50 mL of the previously prepared gold nanoparticle solution containing 9.3 mg AuNPs. The two components were then mixed together at room temperature, and the produced material was then stored in an oven at 60 °C until all the liquid evaporated, as shown in Scheme 1.

The resulting composite material was then characterized using transmission electron microscopy (TEM), scanning electron microscopy (SEM), energy-dispersive X-ray spectroscopy (EDS), Fourier-transform infrared spectroscopy (FTIR) and X-ray diffraction (XRD).

### 2.3. Characterization

For the transmission electron microscopy analysis, a sample of 10 mg of the AuFGLM catalyst was dispersed and sonicated in 5 mL of DI water. Therefore, the final concentration of the composite dispersion prepared for the TEM grid was 2 g per liter. A few drops of the resulting solution were added to a copper grid (300 mesh). Several of these grids were made and dried in an oven at 80 °C overnight. Each grid was then staged and scanned via transmission electron microscopy (TEM, JEM-2100F).

Images of the AuFGLM material were also obtained by scanning electron microscopy (SEM, JEOL JSM-6060LV, Akishima, Tokyo, Japan). This analysis was coupled with energy-dispersive X-ray spectroscopy (EDS, Thermo Scientific UltraDry, Waltham, MA, USA), allowing us to determine the weight ratio of the different elements present within the composite.

The composite’s crystal structures were characterized using powdered X-ray diffraction (P-XRD, Rigaku Miniflex II, Tokyo, Japan), Cu Kα X-ray, nickel filters). Each sample was spread flat on a sample holder and inserted into the instrument which scanned the material from 5° to 90°.

Fourier-transform infrared spectroscopy (FTIR, Shimadzu IR-Tracer 100, Kyoto, Japan) with an attenuated total reflectance attachment (ATR, Shimadzu QATR-S, Kyoto, Japan) was then used to determine any functional groups present in the sample with a scanning range from 4000 cm^−1^ to 500 cm^−1^.

The chemical structure of the AuGLM was also verified by Raman microscope and spectrometer (Renishaw, ISC3-1233, Wotton-under-Edge, UK). The sample was spread flat on the sample slide. The AuFGLM was first scanned at 50× focus from 2000 cm^−1^ to 150 cm^−1^. Then, the microscope was changed to 100× focus to scan the AuNPs on the AuFGLM from 2000 cm^−1^ to 150 cm^−1^.

### 2.4. Catalysis

The setup consisted of two vacuum flasks connected by a simple plastic hose. One flask was designated as the reaction chamber inside of which the hydrolysis reaction of NaBH_4_ catalyzed by the novel AuFGLM catalyst occurred. The second flask contained DI water to be displaced by the hydrogen generated in the first flask. Both reaction chambers were sealed using rubber stoppers; however, the rubber stopper used to seal the second flask, containing the DI water to be displaced, was connected to a cup on a mass balance via a hose that went through the rubber stopper. This mass balance was connected to a computer which recorded the measured mass of the water displaced by the hydrogen gas every 0.25 s. This experiment was run at a variety of pH solutions (6, 7, 8), temperatures (283 K, 288 K, 295 K, 303 K), and NaBH_4_ doses (625 μmoles, 925 μmoles, 1225 μmoles) in order to determine which reaction conditions were optimal. All reactions were stirred using a magnetic stir bar for a full two hours of the trial.

## 3. Results and Discussion

**Figure 1:**
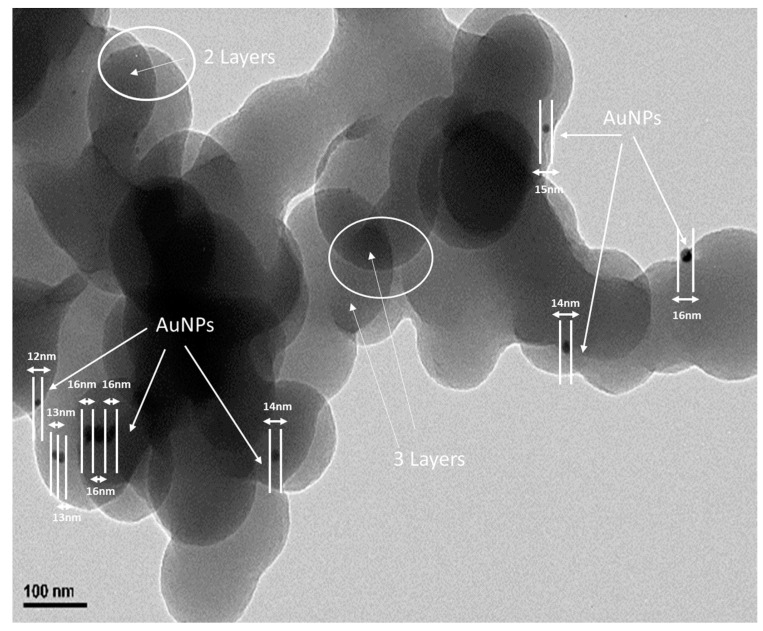
Transmission electron microscopy image of the novel AuFGLM composite catalyst at a scanning range of 100 nm. The white circle depicted the layers of AuFGLM.

TEM micrographs (Figure 1) were used to confirm the presence of nanoparticles supported on t fused graphene-like materials. The image clearly shows the fused carbon backbone as it begins to take on a rounder shape. The material appears to have multiple thin layers stacked upon each other with areas of overlap that appear darker. Dark spherical gold nanoparticles can be seen on the material; the average diameter of the nanoparticles was determined to be 15 nm (±0.5 nm).

**Figure 2:**
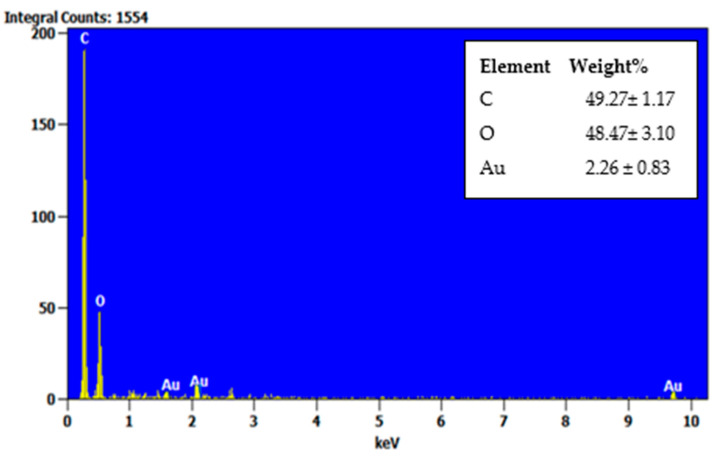
Energy-dispersive X-ray spectroscopy analysis for the novel AuFGLM catalyst.

The AuFGLM catalyst was then characterized using EDS analysis. The EDS analysis (Figure 2) indicated that the AuFGLM contained approximately 49% carbon (wt%), 49% oxygen (wt%), and 2% gold (wt%).

**Figure 3:**
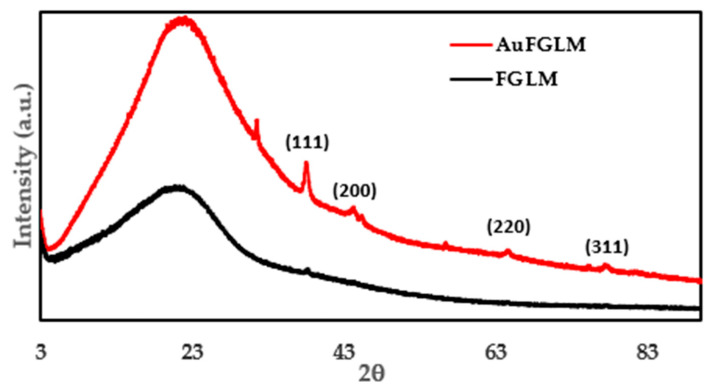
Powder X-ray diffraction patterns of the unsupported fused graphene-like materials and the novel AuFGLM composite.

The low weight percentage of the gold indicated that either the size of the nanoparticles was incredibly small or the gold was very well-dispersed throughout the sample. Due to the low weight percentage, the XRD peaks of the gold nanoparticles in Figure 3 were smaller than that of carbon. The small size of the nanoparticles is supported by the TEM images shown in Figure 1.

The FGLM and AuFGLM materials were also characterized using powder X-ray diffraction (Figure 3). In both materials, a large broad peak can be observed at 23 degrees. This peak is commonly associated with carbon-based materials and is indicative of the fused graphene-like materials [24]. Four peaks that are indicative of gold nanoparticles were seen at 38.2°, 44.5°, 65.3°, and 77.7°. These peaks can, respectively, be attributed to the (111), (200), (220) and (311) lattice planes of the gold nanoparticles present in the material (JCPDS 04-0784) [28].

**Figure 4:**
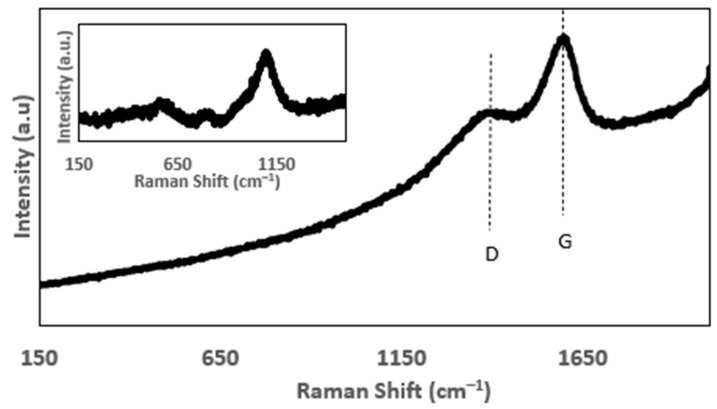
Raman spectrum of AuFGLM at 50× focus. The inset shows the Raman spectrum of AuFGLM at 100× focus. D indicated the D band. G indicated the G band.

Figure 4 showed the Raman spectra of AuFGLM at 50× and 100× focus. At 50× focus, the AuFGLM indicated the G band at 1600 cm^−1^ and D band at 1374 cm^−1^ of the FGLM. A study by Perumbilavil et al. (2015) reported that the G band of graphene materials ranged from 1607 cm^−1^ to 1595 cm^−1^ [29]. The G band occurs as a result of the sp^2^ carbon form. In a study by Lee et al. (2021), the graphene materials showed that the D band ranged from 1330 cm^−1^ to 1380 cm^−1^ [30]. The D band is formed from the defects and disorder in the carbon lattice. Since the AuNPs were well-dispersed on the materials, we turned the microscope to 100× to focus the laser on the AuNPs. The inset in Figure 4 shows that the AuNPs have Raman shifts at 1095 cm^−1^, 784 cm^−1^ and 554 cm^−1^. The results are approximately consistent with many reported studies. For example, a study by Govindaraju et al. (2015) reported that the Raman shifts of gold nanoparticles were found to be between 419 cm^−1^ and 709 cm^−1^ [31]. In a study by Lai et al. (2017), Raman shifts between 1000–1100 cm^−1^ were observed in gold nanoparticles [32].

**Figure 5:**
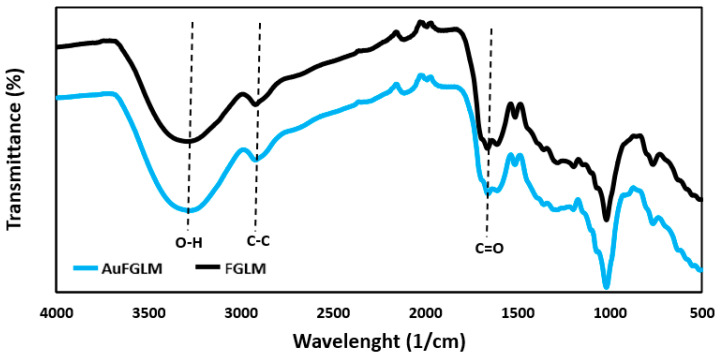
Fourier-transform infrared spectrum of the FGLM support material and the novel AuFGLM composite.

The fused graphene-like material and AuFGLM composite materials were characterized using FTIR (Figure 5). The fused graphene-like material exhibits a long broad peak from roughly 3600 cm^−1^ to 3000 cm^−1^ which is characteristic of a hydroxyl functional group. A simple alkane group is represented by the short peak found at 2900 cm^−1^. A peak seen at 1658 cm^−1^ is indicative of a C=O functional group. All three of these functional groups are characteristic of the dextrose molecule used to synthesize the fused graphene-like material backbone. As expected, all three of these peaks were also seen in the AuFGLM composite with little to no change in intensity, and with small shifts due to the presence of AnNPs. This indicates that the addition of gold nanoparticles to the fused graphene-like material backbone did not significantly alter its structure.

**Figure 6:**
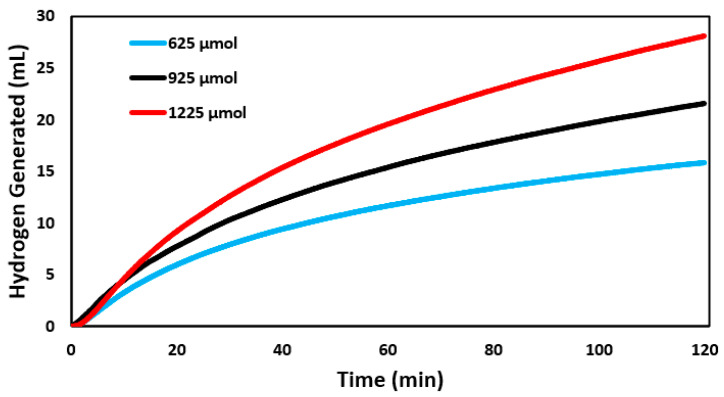
Volume of hydrogen generated over a period of two hours at varying doses of sodium borohydride.

The catalytic efficiency of AuFGLM began by testing its performance at different doses of NaBH_4_ (Figure 6). The catalyst was first tested at a dose of 625 μmol of NaBH_4_, which produced roughly 15.8 mL of hydrogen after a trial time of two hours. The hydrogen generation rate was then calculated to be 0.0132 mL min^−1^ ${\mathrm{mg}}_{\mathrm{cat}}^{-1}$. Increasing the dose of sodium borohydride to 925 μmol resulted in an increased hydrogen generation rate of 0.0180 mL min^−1^ ${\mathrm{mg}}_{\mathrm{cat}}^{-1}$ and a volume of 21.6 mL of hydrogen gas produced. Further increasing the dose to 1225 μmol increased the rate and volume of hydrogen generated even further, at 0.0234 mL min^−1^ ${\mathrm{mg}}_{\mathrm{cat}}^{-1}$ and 28.1 mL, respectively. The data show that there is a direct relationship between the dosage of NaBH_4_ used and the volume of hydrogen generated, which agrees with Le Chatlier’s principle and Equation (3).

**Figure 7:**
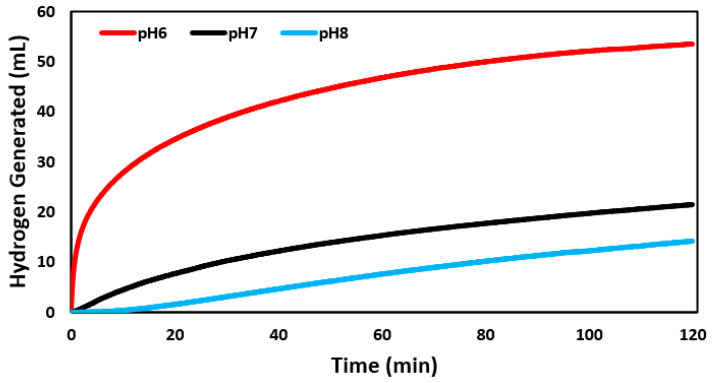
Volume of hydrogen generated over a period of two hours at varying pH solutions.

Next, the catalytic ability of AuFGLM was tested under various pH solutions, as shown in Figure 7. First, the reaction was tested at neutral conditions (pH 7), which produced a hydrogen gas volume of 21.6 mL at a rate of 0.0180 mL min^−1^${\mathrm{mg}}_{\mathrm{cat}}^{-1}$. The pH was then lowered to pH 6 which resulted in over twice as much hydrogen gas, with 53.5 mL produced at a rate of 0.0446 mL min^−1^${\mathrm{mg}}_{\mathrm{cat}}^{-1}$. When the pH of the reaction was raised to pH 8, it was observed that the rate of hydrogen generation had decreased to 0.0118 mL min^−1^${\mathrm{mg}}_{\mathrm{cat}}^{-1}$, producing only 14.1 mL of hydrogen gas after two hours. Based on this data, it was determined that the reaction catalyzed by AuFGLM performed better under acidic conditions. This phenomenon was previously reported in work by Schlesinger et al. (1953) who found that stronger acids sped up the hydrogen generation of this reaction, possibly due to an increase in the free hydrogen ions present in the solution [3]. Inversely, increasing the pH of the reaction was observed to slow the rate of generation, as was observed by Kaufman et. al. (1985) and also by Grzeschik et al. (2020), and in previous work by this research team [11,12,13,14,33,34,35,36,37]. It may be that hydroxide ions compete for hydrogen in the solution.

**Figure 8:**
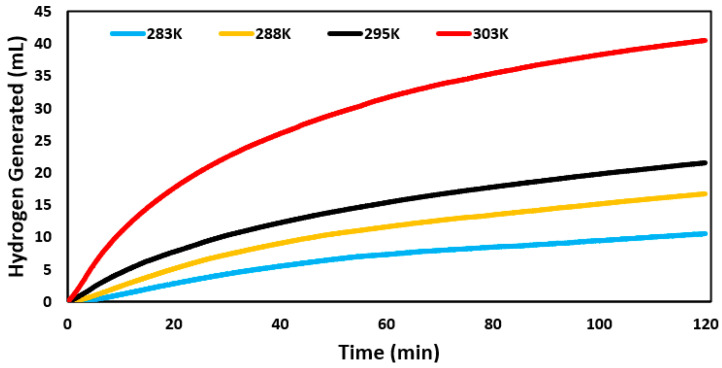
Volume of hydrogen generated over a period of two hours at varying solution temperatures.

The AuFGLM was then tested at varying temperatures (Figure 8).

For the temperature trial, the temperature of the reaction was cooled to 283 K and produced 10.6 mL of hydrogen gas at a rate of 0.0088 mL min^−1^${\mathrm{mg}}_{\mathrm{cat}}^{-1}$. At a temperature of 288 K, the reaction produced hydrogen gas at a rate of 0.0139 mL min^−1^ ${\mathrm{mg}}_{\mathrm{cat}}^{-1}$ and 16.7 mL after two hours. At room temperature (295 K), the reaction produced 21.6 mL of hydrogen gas at a rate of 0.0180 mL min^−1^${\mathrm{mg}}_{\mathrm{cat}}^{-1}$. Finally, when the temperature of the reaction was heated to 303 K an increase in hydrogen gas generation was observed, with 40.5 mL produced at a rate of 0.0337 mL min^−1^${\mathrm{mg}}_{\mathrm{cat}}^{-1}$. Based on these conditions it is clear that there is a positive linear relationship, with increases in temperature resulting in increases in hydrogen gas generation. According to Le Chatlier’s principle, this means that the reaction is endothermic.

Once the temperature trials had been completed, data could be plugged into the Arrhenius Equation (2) to find the rate constant (k) at each temperature. In the following equation the variables are represented as the following; k is the rate constant of the reaction at the tested temperature (T) in Kelvin. The variable A represents the pre-exponential factor, and the variable R is the universal gas constant. Lastly, Ea represents the activation energy of the reaction.

(2) $$k={\mathrm{Ae}}^{-\frac{\mathrm{Ea}}{\mathrm{RT}}}$$

**Figure 9:**
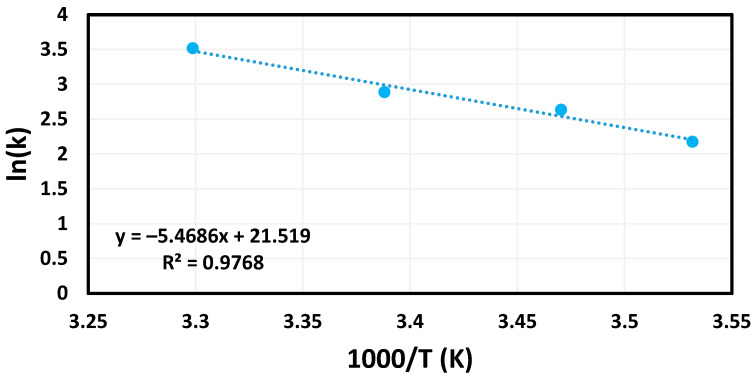
Arrhenius plot created by the using the Arrhenius Equation (3) and the temperature of the trials. The blue dot line indicated the trendline.

Using the natural log of the rate constant found at each temperature vs. that temperature divided by 1000 (Figure 9), the slope of the line allowed for the activation energy of AuFGLM to be determined to be 45.5 kJ mol^−1^.

The activation energy of this catalyst was then compared to similar catalysts for the hydrolysis of NaBH_4_, as seen in Table 1.

As shown in Table 1, AuFGLM has an activation energy that is competitive compared to other catalysts for the hydrolysis reaction of sodium borohydride. Nanoporous graphene oxide (PGO), a material that is similar to our unsupported FGLM, showed no ability to produce hydrogen in this reaction [42]. As such, FGLM on its own was not believed to be able to produce hydrogen on its own. When compared to bulk non-precious metal catalysts such as nickel and cobalt, AuFGLM had a significantly lower activation energy. Compared to unsupported gold nanoparticles, the AuFGLM again had a lower activation energy, indicating that the addition of the fused graphene-like material support aided in the gold nanoparticle’s catalytic ability. Unsupported platinum nanoparticles, however, outperformed the composite, possibly hinting at platinum being a better catalyst than gold; however, this is just speculation, and more work needs to be done to confirm this. Other carbon-supported catalysts either performed better or worse than the AuFGLM, depending on the metal, again indicating that more research is needed to improve our understanding of which catalysts are best for this reaction.

**Figure 10:**
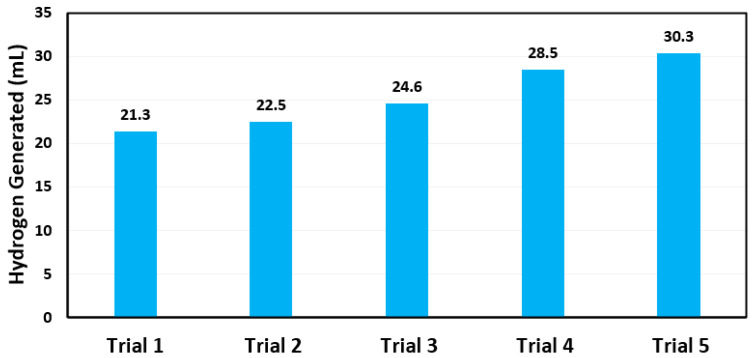
Volume of hydrogen gas produced for five consecutive two-hour trials in standard conditions.

The final catalytic study conducted concerned the reusability of the catalyst. A trial was set up with standard conditions (295 K, pH 7, and a NaBH_4_ dose of 925 μmol) that ran for two hours. After the two-hour period was completed, an additional 925 μmol dose was added to the same reaction vessel, marking the start of the second trial. This was repeated for a total of five trials, producing an average volume of 25.4 mL per two-hour trial, or 28% based on the theoretical maximum of 90 mL (Figure 10). The data show that the catalyst produced similar amounts of hydrogen for three consecutive trials, after which there was an increase in hydrogen generation. The increasing trend of the hydrogen generation is related to the binding of BH_4_^−^ and H^−^ species on the surface of gold nanoparticles, as shown in Equation (3). Over time, these bonds became hydrolyzed, as shown in Equation (4) and improved the electrostatic stabilization of the AuNPs surface, making them more active [43]. These results not only indicate that the catalyst is stable and can be reused, cutting costs and materials, but also could mean that the catalyst becomes more catalytically activated with multiple uses.

2 Surface-Au + BH_4_^−^ ↔ Surface-Au-H + Surface-Au-BH_3_^−^ (3)

Surface-Au-H + Surface-Au-BH_3_^−^+ HOH → Surface-Au + H_2_ + [BH_2_(OH)]^−^ (4)

**Scheme 2:**
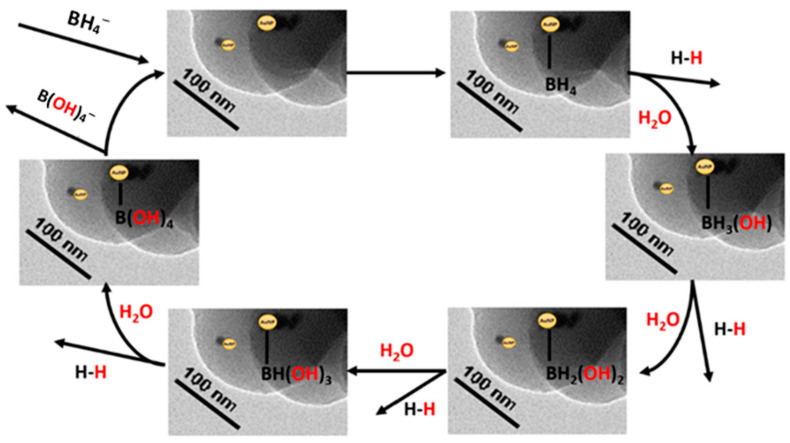
Proposed mechanism for the catalytic hydrolysis of NaBH_4_.

Scheme 2 depicts one possible mechanism for the catalytic hydrolysis of NaBH_4_ by AuFGLM. The gold nanoparticles are supported on the surface of the fused graphene-like material. In the solution, the NaBH_4_ dissociated into borohydride ions (BH_4_)^−^ which then bonded with the nanoparticles. Water in the solution then attacked the boron in the borohydride and split off one hydrogen atom. This hydrogen atom bonded with a hydrogen atom from the BH_4_, which then released itself as a diatomic hydrogen molecule. The remaining hydroxyl (OH) group from the water stayed fixed to the boron atom. This happened four times in total, at which point the resulting tetrahydroxyborate [B(OH)_4_^−^] molecule left the nanoparticle and another borohydride ion took its place to restart the cycle.

## 4. Conclusions

Fused graphene-like materials (FGLMs) have a structure that combines multiple graphene-like layers into a single cohesive material. The specific combination of different graphene-like layers can be tailored to achieve desired functionalities as a catalyst support. The FGLMs supporting the gold nanoparticles were characterized via TEM in order to determine the general morphology of the composite and the adhesive properties of the nanoparticles. The catalytic properties of the AuFGLM composite were tested via a hydrogen evolution reaction involving NaBH_4_, where it was found that the optimal conditions for AuFGLM to produce hydrogen are increased temperatures, increased NaBH_4_ doses, and decreased pH’s. This catalyst produced the most hydrogen at a pH value of 6, at a rate of 0.0446 mL min^−1^${\mathrm{mg}}_{\mathrm{cat}}^{-1}$. Temperature data allowed the activation energy of this reaction as catalyzed by the AuFGLM to be 45.5 kJ mol^−1^, which makes it competitive compared to other similar catalysts for this reaction. Additionally, it was found that the catalyst was stable for up to at least five consecutive trials, producing an average hydrogen volume of 25.4 mL. This stability, along with a competitive activation energy, makes this catalyst a suitable option for optimizing the hydrolysis of NaBH_4_ for the generation of hydrogen as an alternative fuel source.

## Figures and Tables

**Table 1:** Comparison of activation energies.

Catalyst	E_a_ (kJ mol^−1^)	Temperature (K)	Reference
Ni	71	273–308	[33]
Raney-Nickel	63	273–308	[33]
Co	75	273–308	[33]
Ni–Co–B	62	281–301	[36]
Ru/ZIF	36.4	303–333	[37]
Ru-on-carbon	66.9	298–358	[38]
Pt/MWCNTs	46.2	283–303	[34]
Ag/MWCNTs	44.5	273–303	[12]
Pd/MWCNTs	62.7	273–303	[14]
Au/MWCNTs	21.1	273–303	[18]
PtNPs	39.2	283–303	[13]
BCD-AuNP	54.7	283–303	[11]
Pd Nanocup	58.9	283–303	[39]
AgNPs	50.3	273–303	[40]
PtNPs	50.3	273–303	[41]
PGO	N/A	298	[42]
AuFGLM	45.5	283–303	This Work

## Data Availability

The data presented in this study are available on request from the corresponding author.
